# Association between visceral adiposity index and endometriosis: a population-based study

**DOI:** 10.3389/fnut.2025.1602288

**Published:** 2025-07-21

**Authors:** Jiajia Zhang, Qian Zhang, Tianyu Chu, Xian Chen, Hui Zhou, Dewu Xu, Chunlin Dong, Yibo Wu

**Affiliations:** ^1^Obstetrics, Gynecology and Reproduction Research Center, Affiliated Hospital of Jiangnan University, Wuxi, China; ^2^Department of Medical Education, Affiliated Hospital of Jiangnan University, Wuxi, China

**Keywords:** endometriosis, visceral adiposity index, obesity, NHANES, cross-sectional survey

## Abstract

**Objectives:**

Obesity and endometriosis are intricately linked. The body mass index (BMI) is commonly used as an indicator of obesity, but it has limitations. The visceral adiposity index (VAI) is a novel, low-cost composite index that reflects visceral adiposity accumulation and metabolic health status. The objective of our research was to investigate the association between VAI and endometriosis.

**Methods:**

This is a cross-sectional study. The investigation utilized information from the NHANES (1999–2006), focusing on participants aged ≥20 years. We evaluated the association between VAI and endometriosis through five complementary approaches: multivariable-adjusted weighted logistic regression, trend tests, sensitivity analyses, subgroup analyses, and restricted cubic spline (RCS) curve.

**Results:**

After screening, our analysis included 2,056 eligible subjects, among whom 163 cases of endometriosis were identified. The final adjusted logistic regression model demonstrated a significant positive association between VAI and endometriosis (OR = 1.08, 95% CI: 1.04–1.12, *p* < 0.001). Results of restricted cubic spline fitting revealed a linear positive correlation between VAI and endometriosis (*p* for overall < 0.001; *p* for non-linear = 0.539). The results of subgroup analyses showed that some specific demographic, lifestyle, and reproductive characteristics were not statistically significant in influencing the correlation between VAI and endometriosis (*p* > 0.05 for all interactions).

**Conclusion:**

Our study observed a statistically significant association between VAI and endometriosis. More prospective cohort investigations with large samples are required to further validate these findings because the etiology of endometriosis remains unclear.

## 1 Introduction

Endometriosis (EMS) is the appearance of endometrial organizations (glands and mesenchyme) with growth function in sites other than the uterine cavity (1). The endometrium in an abnormal position can implant anywhere in the body, such as the umbilicus, urinary tract, lungs, and conjunctiva; however, the most common sites are the pelvis, ovaries, and uterosacral ligaments. Other frequent locations include the pelvic peritoneum and the rectovaginal septum (2). Endometriosis is often accompanied by dysmenorrhea, infertility, and a variety of emotional disorders, which can seriously affect women’s reproductive and psychological health (3). Multiple studies have demonstrated that obesity significantly alters the risk profile for several gynecological conditions, including infertility, endometriosis, abnormal uterine bleeding, and polycystic ovary syndrome (4). With the development of the economy and changes in dietary structure, nutritional levels, and lifestyles, obesity has become a globally recognized major health problem (5, 6). CT and MRI are associated with radiation exposure, high cost, and time-consuming procedures. As a result, most primary care patients refuse to undergo these and other screening methods that can accurately measure body fat, primarily due to healthcare setting limitations, financial constraints, and other factors. Body mass index (BMI) is often utilized to evaluate obesity in individuals. However, some studies have shown that BMI has limited diagnostic properties. It does not distinguish between muscle and lipid mass, does not identify individuals with excess body fat, and does not accurately characterize fat distribution (7, 8). Although some existing studies have analyzed the connection between BMI and EMS, there is a slight inconsistency in their conclusions (9). In addition, current studies on the relationship between obesity, fat distribution, and EMS are insufficient, highlighting the need for further research (10).

Studies have shown that elucidating the intrinsic association between endometriosis and obesity is a challenging but promising research topic. Pantelis et al. suggested that future studies could focus on different anthropometric measures and alternative methods to elucidate the complex relationship between obesity and endometriosis (10). It has been suggested that adipose tissue function is strongly associated with endometriosis (11). Due to the distinct characteristics of adipose tissue distribution and the gaps in current research, this study aimed to explore the correlation between visceral fat and endometriosis.

In 2010, Amato et al. proposed a gender-integrated personalized indicator of visceral obesity (12). The visceral adiposity index (VAI) is a composite index calculated from waist circumference (WC), body mass index (BMI), high-density lipoprotein cholesterol (HDL-C), and triglycerides (TG) (13). VAI is capable of assessing visceral adiposity distribution and metabolic impairment, and it has the potential for risk prediction in infertility, cardiovascular disease, diabetes, and other diseases (14–16). Nevertheless, there are very few systematic studies on the correlation between VAI and endometriosis in the population.

Therefore, our study analyzed the potential relationship between visceral fat accumulation and endometriosis using data from the National Health and Nutrition Examination Survey (NHANES), a large-scale database. Our study aimed to provide theoretical references for the early prediction and risk assessment of endometriosis.

## 2 Materials and methods

### 2.1 Study design and participants

NHANES is a publicly available database of cross-sectional surveys conducted throughout the United States. A stratified, multi-stage random sampling method was used to ensure that the sample was nationally representative (17). The data collection process was subject to strict quality control. The investigators were professionally trained and assessed, and they followed a standardized protocol to conduct questionnaires, physical examinations, and laboratory tests, as well as to collect information on the study participants. Each year, approximately 5,000 participants are recruited into this study from all regions of the United States. NHANES contains not only traditional physical exam and laboratory test data but also incorporates detailed dietary, lifestyle, and socioeconomic background information, providing researchers with a valuable resource for exploring the complex relationship between health and nutrition. The National Center for Health Statistics (NCHS) Ethics Review Board (ERB) approved each NHANES study protocol, and each participant provided written informed consent.

**Figure 1:**
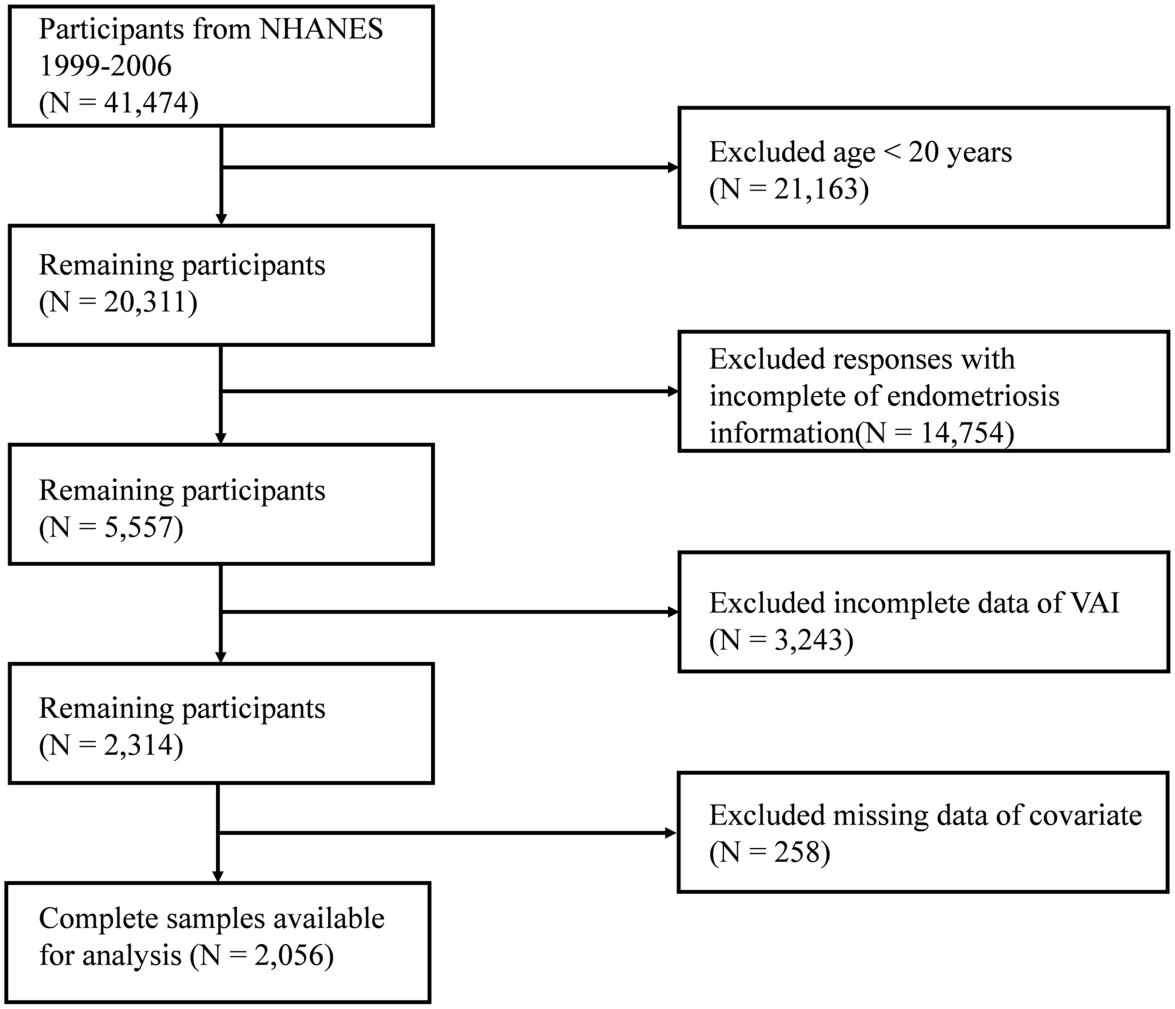
Flowchart of the inclusion and exclusion of study participants.

Because of the availability of comprehensive data on endometriosis, participants from 1999 to 2006 were included in our study. Initially, 41,474 participants were included. After excluding individuals aged <20 years (*n* = 21,163), those who did not have complete information on endometriosis (*n* = 14,754), those with missing information related to VAI (*n* = 3,243), and those with missing information on covariates (*n* = 258), data from 2,056 participants were retained for statistical analysis (Figure 1).

### 2.2 Calculation of VAI

VAI is a sex-specific composite indicator calculated from WC, BMI, TG, and HDL-C to evaluate the degree of visceral fat accumulation (12). The following formula is used to calculate it. In the formula, BMI is expressed in kg/m^2^, WC in cm, and HDL-C and TG in mmol/L. Higher VAI levels represent more severe levels of visceral obesity.


$$\mathrm{VAI}(\text{male})=(\frac{\mathrm{WC}}{39.68+1.88*\mathrm{BMI}})*(\frac{\mathrm{TG}}{1.03})*(\frac{1.31}{\mathrm{HDL}-C})$$

$$\mathrm{VAI}(\text{female})=(\frac{\mathrm{WC}}{36.58+1.89*\mathrm{BMI}})*(\frac{\mathrm{TG}}{0.81})*(\frac{1.52}{\mathrm{HDL}-C})$$

### 2.3 Endometriosis

Endometriosis diagnostic information was extracted from self-reported questionnaire data in NHANES. The questionnaire variable is called RHQ360. Participants were asked if they had been diagnosed with endometriosis by a healthcare professional, and only those with clear results were included in the study. Participants were categorized as having endometriosis if they answered “yes,” and as not having endometriosis if they answered “no.” Diagnosis of endometriosis based on self-report has some limitations. Potential underdiagnosis or misclassification may occur, and there is no way to account for the severity of the disease or the patient’s laparoscopic information. However, studies have shown that patients’ recall of their endometriosis history can be more than 70% accurate (18). The feasibility of screening for endometriosis using self-reported data has also been confirmed by several studies based on the NHANES database (19, 20).

### 2.4 Covariates

The covariates were selected based on clinical experience and previous studies (21–23). Covariates in our study included the following: Demographic variables: age, ethnicity (Mexican American, other Hispanic, non-Hispanic White, non-Hispanic Black, other ethnicities), educational level (less than high school, high school or GED, above high school), marital status (married/living with a partner, never married, widowed/divorced/separated), and poverty–income ratio (PIR: <1.3, 1.3–3.5, >3.5); lifestyle factors: smoking (no/yes), drinking (no/yes) and physical activity (vigorous/ moderate/ sedentary); reproductive characteristics: use of oral contraceptives (no/yes), pregnancy history (no/yes), and age at menarche (<12, ≥12); and clinical variables: diabetes (no/yes) and hypertension (no/yes). Smoking was judged by the question “Smoked at least 100 cigarettes in life?” Drinking was categorized by the question “Had at least 12 alcohol drinks in 1 year?” Diabetes and hypertension were determined by participants’ self-reported outcomes.

### 2.5 Statistical analysis

To ensure that the investigation data truly and comprehensively reflect the actual situation nationwide, the study adopted an analytical method combining sampling weights and complex sample design. This method effectively corrected the problem of possible over-representation of certain specific groups in the sample, thereby avoiding bias in the results that could arise from an uneven sample distribution. Data analysis was implemented in R software (version 4.4.3). A significance threshold of *p* < 0.05 was applied to all inferential tests.

Continuous variables were described using weighted means and standard errors, and categorical variables were described using numbers and weighted percentages. When comparing baseline characteristics between groups, we used weighted Student’s t-tests and weighted chi-squared tests. Following descriptive analyses, weighted multifactorial logistic regression was used to determine the connection between VAI and endometriosis. Before constructing the multivariate model, we assessed multicollinearity among the variables. Model 1 was a basic model we constructed, considering only VAI. Model 2 adjusted for age, education level, race, marital status, and PIR. Model 3 further adjusted for smoking, alcohol consumption, diabetes, hypertension, use of oral contraceptives, pregnancy history, age at menarche, and physical activity, based on Model 2. In addition, we performed trend tests for VAI and EMS. We used a restricted cubic spline (RCS) curve to further analyze the correlation between VAI and EMS. We identified three knots (located at the 10th, 50th, and 90th percentiles of VAI) to provide the best fit according to the minimum Akaike information criterion (AIC). Sensitivity analyses were conducted to determine the robustness of the results. Participants with hypertension and diabetes were excluded, and covariates were adjusted stepwise in the statistical modeling. Finally, we conducted subgroup analyses to explore the presence of confounders influencing the association between VAI and endometriosis.

## 3 Results

### 3.1 Baseline characteristics

There were 2,056 individuals in our study, including 163 participants diagnosed with endometriosis and 1,893 participants without endometriosis. The endometriosis group had a higher mean age (40.20 ± 8.21 years) compared to the control group (37.26 ± 10.01 years). In this study population, the VAI quartiles were categorized as follows: Quartile 1 (<0.931), Quartile 2 (0.931–1.477), Quartile 3 (1.477–2.406), and Quartile 4 (≥2.406). We detected significant differences between endometriosis patients and non-endometriosis patients in terms of age, race, education level, whether or not they smoked, use of oral contraceptives, history of pregnancy, triglyceride (TG) levels, and VAI (*p* < 0.05). More baseline characterization information is presented in Table 1.

**Table 1:** Weighted demographic characteristics of all participants.

Participant characteristic	Non-endometriosis (*N* = 1893)	Endometriosis (*N* = 163)	*p*-value
Age (years)	37.26 ± 10.01	40.20 ± 8.21	<0.001
Ethnicity			<0.001
Mexican American	463 (8.25%)	9 (1.40%)	
Other Hispanic	79 (5.40%)	5 (1.94%)	
Non-Hispanic White	877 (68.55%)	114 (84.51%)	
Non-Hispanic Black	385 (12.31%)	30 (8.29%)	
Other race	89 (5.49%)	5 (3.86%)	
Education level			0.008
Less than high school	452 (15.94%)	19 (10.24%)	
High school or GED	391 (21.73%)	44 (31.74%)	
Above high school	1,050 (62.33%)	100 (58.03%)	
Marital status			0.050
Married/living with a partner	1,284 (67.40%)	111 (74.88%)	
Never married	347 (17.87%)	22 (8.67%)	
Widowed/divorced/separated	262 (14.73%)	30 (16.45%)	
Poverty–income ratio (PIR)			0.593
<1.3	527 (20.75%)	37 (21.47%)	
1.3–3.5	706 (35.84%)	54 (30.48%)	
>3.5	660 (43.41%)	72 (48.05%)	
Smoking			<0.001
No	1,206 (59.45%)	77 (40.37%)	
Yes	687 (40.55%)	86 (59.63%)	
Drinking			0.585
No	745 (31.97%)	51 (29.19%)	
Yes	1,148 (68.03%)	112 (70.81%)	
Oral contraceptive			0.005
No	437 (20.07%)	15 (8.91%)	
Yes	1,456 (79.93%)	148 (91.09%)	
Pregnancy history			0.047
No	263 (18.08%)	21 (12.55%)	
Yes	1,630 (81.92%)	142 (87.45%)	
Age at menarche (years)			0.327
<12	435 (21.83%)	44 (25.81%)	
≥12	1,458 (78.17%)	119 (74.19%)	
Diabetes			0.636
No	1818 (96.11%)	157 (96.84%)	
Yes	75 (3.89%)	6 (3.16%)	
Hypertension			0.451
No	1,560 (81.17%)	119 (78.80%)	
Yes	333 (18.83%)	44 (21.20%)	
Physical activity			0.411
Vigorous	600 (37.59%)	59 (36.81%)	
Moderate	580 (31.46%)	43 (27.24%)	
Sedentary	713 (30.95%)	61 (35.94%)	
WC (cm)	91.99 ± 16.07	94.04 ± 15.14	0.083
BMI (kg/m^2^)	28.04 ± 7.19	28.45 ± 6.55	0.248
TG (mmol/L)	1.32 ± 0.93	1.91 ± 2.55	<0.001
HDL-C (mmol/L)	1.49 ± 0.41	1.45 ± 0.43	0.297
VAI	1.99 ± 2.14	3.32 ± 6.39	<0.001

The numbers of participants in each category are unweighted observed frequencies, while means, standard errors, and percentages are population-weighted.

### 3.2 Correlation between VAI and endometriosis

**Figure 2:**
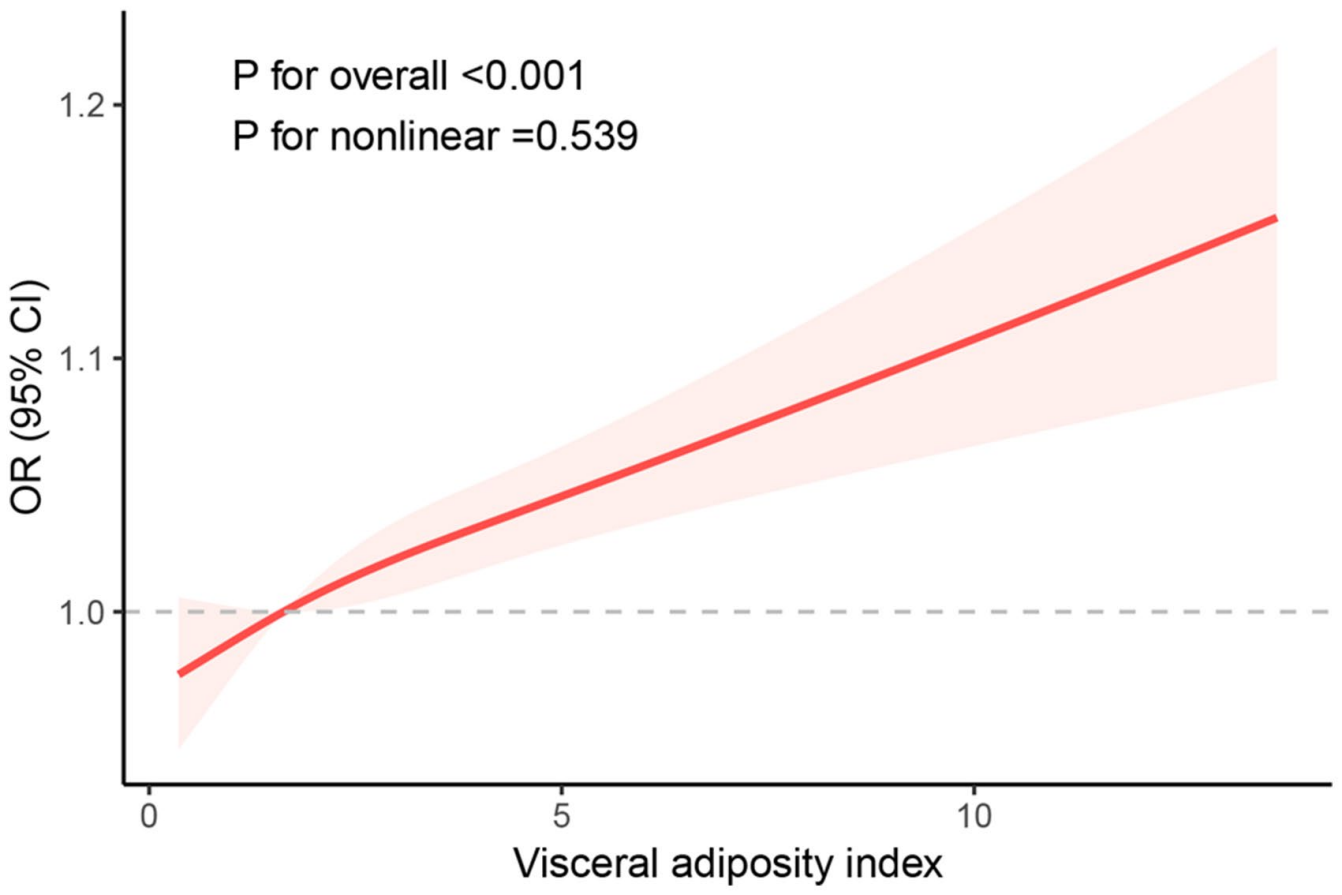
RCS curve of the association between the VAI index and endometriosis among all study participants.

We conducted weighted univariate and multivariate regression analyses, as shown in Table 2. The variance inflation factors (VIFs) for all covariates included in the study were less than 10, indicating no significant multicollinearity among the variables (Supplementary Table S1). We also evaluated the association between BMI and WC with endometriosis separately (Supplementary Table S2). Our findings suggested that a higher degree of VAI was related to an increased risk of EMS. In the crude model, VAI was positively correlated with endometriosis with an OR of 1.10 (95% CI: 1.05–1.15, *p* < 0.001). In the model partially adjusted for demographic variables, VAI was positively associated with endometriosis, with an OR of 1.09 (95% CI: 1.05–1.13, *p* < 0.001). In the model adjusted for all covariates, this correlation remained significant with an OR of 1.08 (95% CI: 1.04–1.12, *p* < 0.001). Based on the above findings, we categorized the participants into Q1, Q2, Q3, and Q4 groups based on the quartiles of VAI to further explore the differences in endometriosis risk among them. In the unadjusted model, women in Q4 exhibited a 2.09-fold increased risk of disease compared to those in the Q1 group (95% CI: 1.27–3.43, *p* = 0.004). In the partially adjusted model, women in Q4 exhibited a 1.95-fold increased risk of disease compared to those in the Q1 group (95% CI: 1.17–3.27, *p* = 0.012). In the fully adjusted model, women in Q4 exhibited a 1.78-fold increased risk of disease compared to those in the Q1 group (95% CI: 1.08–2.96, *p* = 0.026). In all models, a significant increasing trend in the quartiles of VAI with respect to the odds of EMS was observed. (all *p* for trend < 0.05). Furthermore, RCS curves showed a linear positive correlation between VAI and EMS (Figure 2).

**Table 2:** Association between VAI index and the risks of endometriosis.

VAI	OR (95%CI), *p*-value		
	Crude model	Minimally adjusted model	Fully adjusted model
	(Model 1)	(Model 2)	(Model 3)
VAI index	1.10 (1.05, 1.15), *p* < 0.001	1.09 (1.05, 1.13), *p* < 0.001	1.08 (1.04, 1.12), *p* < 0.001
VAI index (quartile)			
Q1	Reference	Reference	Reference
Q2	1.34 (0.78, 2.31), *p* = 0.283	1.32 (0.77, 2.25), *p* = 0.302	1.28 (0.73, 2.22), *p* = 0.378
Q3	1.39 (0.83, 2.32), *p* = 0.207	1.37 (0.80, 2.33), *p* = 0.247	1.25 (0.73, 2.12), *p* = 0.408
Q4	2.09 (1.27, 3.43), *p* = 0.004	1.95 (1.17, 3.27), *p* = 0.012	1.78 (1.08, 2.96), *p* = 0.026
*p* for trend	0.005	0.012	0.026

Model 1 adjusted for: none. Model 2 adjusted for: age, race, education, marital status, and PIR. Model 3 adjusted for: age, ethnicity, education, marital status, PIR, smoking, drinking, diabetes, hypertension, oral contraceptive, pregnancy, age at menarche, and physical activity. 95% CI, 95% confidence interval; OR, odds ratio.

### 3.3 Subgroup analysis

In order to determine whether the connection between VAI and EMS was influenced by population-specific factors, we conducted subgroup analyses. Participants were stratified into groups based on their demographic characteristics and health behaviors. In Table 3, the outcome of the analysis demonstrated that the correlation between VAI and endometriosis was consistent across all categories. There was no evidence that factors such as education level, PIR, marital status, age, race, use of oral contraceptives, age at menarche, history of pregnancy, smoking, drinking, hypertension, and diabetes altered the connection between VAI and EMS (*p* > 0.05 for all interactions).

**Table 3:** Subgroup analyses of the effect of VAI on endometriosis.

Subgroup	Category	Total (*N*)	EMS (*N*)	OR (95%CI)	*p* for interaction
Age (years)					0.477
	<50	1819	138	1.05 (1.01, 1.10)	
	≥50	237	25	1.10 (0.99, 1.23)	
Ethnicity					0.382
	Mexican American	472	9	0.87 (0.57, 1.33)	
	Other Hispanic	84	5	1.22 (0.63, 2.35)	
	Non-Hispanic White	991	114	1.11 (1.04, 1.18)	
	Non-Hispanic Black	415	30	1.09 (0.83, 1.44)	
	Other ethnicities	94	5	0.99 (0.75, 1.31)	
Education level					0.443
	Less than high school	471	19	0.99 (0.85, 1.16)	
	High school or GED	435	44	1.08 (1.00, 1.17)	
	Above high school	1,150	100	1.13 (1.04, 1.23)	
Poverty–income ratio (PIR)					0.921
	<1.3	564	37	1.08 (1.01, 1.14)	
	1.3–3.5	760	54	0.98 (0.82, 1.17)	
	>3.5	732	72	1.07 (1.00, 1.15)	
Marital status					0.222
	Married/Living with a partner	1,395	111	1.05 (1.00, 1.11)	
	Never married	369	22	0.79 (0.49, 1.25)	
	Widowed/Divorced/Separated	292	30	1.12 (1.00, 1.26)	
Smoking					0.939
	No	1,283	77	1.05 (0.92, 1.19)	
	Yes	773	86	1.05 (1.01, 1.10)	
Drinking					0.408
	No	796	51	1.09 (1.01, 1.18)	
	Yes	1,260	112	1.05 (1.00, 1.10)	
Oral contraceptive					0.902
	No	452	15	1.07 (0.94, 1.22)	
	Yes	1,604	148	1.06 (1.02, 1.11)	
Pregnancy history					0.291
	No	284	21	1.13 (0.99, 1.30)	
	Yes	1772	142	1.05 (1.01, 1.10)	
Age at menarche (years)					0.505
	<12	479	44	1.09 (1.00, 1.19)	
	≥12	1,577	119	1.05 (1.01, 1.10)	
Diabetes					0.211
	No	1975	157	1.05 (1.01, 1.10)	
	Yes	81	6	1.23 (0.96, 1.57)	
Hypertension					0.509
	No	1,679	119	1.06 (1.01, 1.10)	
	Yes	377	44	1.11 (0.97, 1.28)	

### 3.4 Sensitivity analysis

The results of the sensitivity analysis showed that after excluding participants with hypertension and diabetes, a significant association between VAI and endometriosis remained in the fully adjusted model (OR = 1.05, 95% CI: 1.01–1.09, *p* = 0.016) (Supplementary Table S3). This is consistent with the results of the main study and illustrates the robustness of the findings.

## 4 Discussion

In our research, we explored the relationship between VAI and endometriosis using participants from four cycles of the NHANES database as study subjects. Our study found that VAI was positively and linearly associated with endometriosis. This correlation was mainly independent of confounding variables, including age, use of oral contraceptives, age at menarche, history of pregnancy, race, PIR, hypertension, and diabetes.

Our study is the first to leverage the NHANES database to report the correlation between VAI and EMS. EMS, a complex chronic disease, may involve the combined effects of multiple systems, including endocrine dysfunction, genetics, immunomodulatory abnormalities, and environmental exposures, in its pathogenesis (24). Collectively, the available findings suggested that there are associations between obesity and endometriosis; however, the relationship is complex and not yet fully defined. BMI is commonly used as an indicator of an individual’s obesity. The majority of studies have shown a negative correlation between BMI and endometriosis in women (9, 25). Participants with EMS often have lower BMI and upper arm muscle mass than women without EMS (26). However, the study has shown that endometriosis is not consistently positively correlated with obesity and that this relationship is rare among female reproductive disorders (4). There may be multiple explanations for our findings that individuals with a high VAI are at a high risk of endometriosis. Perhaps this is because VAI serves as a comprehensive assessment indicator that effectively reflects abnormal changes in lipid metabolism, insulin sensitivity, and inflammatory status in individuals (27, 28). As such, it has good efficacy in reflecting the relationship between abnormal metabolic characteristics and EMS. One study analyzed the relationship between the CMI index, which consists of parameters related to lipid and obesity, and EMS. Elevated CMI levels showed a significant association with increased endometriosis risk (23), which is similar to our findings. The difference between the two results is that individuals diagnosed with endometriosis showed several significant differences in characteristics compared to those without endometriosis, such as smoking status and poverty–income ratio (PIR). This may be due to differences in the characteristics of the study population resulting from different inclusion criteria.

However, no correlation between BMI and WC and endometriosis was found in our study population, which is similar to the results of some studies (9). This may be due to sample size limitations and other confounding factors. In the context of endometriosis, BMI indicators in the female population exhibit significant dynamic changes in characteristics. In women with normal BMI but who may have metabolic disturbances due to abnormal fat distribution, the effect of obesity on endometriosis cannot be accurately measured by BMI alone. Waist circumference does not distinguish between subcutaneous and visceral fat mass (29). Visceral adipose tissue function is strongly associated with endometriosis (30). In addition, the VAI is a composite index that takes into account gender specificity, reflecting physiologic differences in fat distribution between men and women, and may be more advantageous in studies of female-associated diseases.

In fact, endometriosis is a chronic inflammatory condition associated with abnormal lipid metabolism (31, 32). Obesity has been shown to affect lipid metabolism through the NF-κB signaling pathway (33). In the inflammatory microenvironment, lipid homeostasis in adipose tissue is disrupted, accompanied by the abnormal aggregation of pro-inflammatory immune cells and a significant increase in the concentration of inflammatory mediators (23). Studies have shown that patients with endometriosis develop high levels of IL-1β, IL-8, IL-6, and other pro-inflammatory factors (34). Aberrant activation of the NF-κB pathway induces the growth and progression of EMS (35). Therefore, it was hypothesized that the association between VAI and EMS might be related to the NF-κB pathway. Additionally, dyslipidemia has been shown to lead to the accumulation and activation of several inflammatory factors, which in turn promote the growth of intraperitoneal inflammation and endometrial lesions (36). TG levels are higher in endometriotic lesions and exacerbate the course of EMS through an inflammatory response (35, 37). Visceral adipose tissue is regulated by endometriosis-derived pro-inflammatory factors to undergo browning (30). Ectopic growth of endometrial tissue directly triggers a localized inflammatory response, which in turn causes an increase in pro-inflammatory cytokines in the peritoneal fluid, such as interleukin-6 and interleukin-33 (38, 39). At the same time, these pro-inflammatory factors in turn contribute to the dysfunction of visceral adipose tissue. Abnormally functioning visceral adipose tissue can further exacerbate the development of endometriosis (30). Some studies have shown that liver lipid synthesis and lipoprotein lipase activity may be inhibited in patients with endometriosis, resulting in triglyceride (TG) dysregulation (40). These may explain the significant correlation between VAI and EMS.

The strengths of our current survey include the utilization of the NHANES database and the consideration of sample weights for sampling, which enhances the representativeness of the results. In addition, we constructed several models to progressively adjust for confounders and conducted trend tests and subgroup analyses to bolster the credibility of our results. When interpreting the results of our research, certain limitations must be considered. The cross-sectional design was unable to determine the causal relationship between VAI and EMS and to explore the dynamic association between VAI and EMS. Some of the information used was obtained from questionnaires addressed to participants and may be subject to bias. The questionnaire on endometriosis in the NHANES database was only collected before 2006, which may impact the timely nature of the study results. In addition, VAI, as a biomarker, has lipid-dependent levels that are influenced by fluctuations in lipid level measurements. Moreover, this study was conducted only in a group of American women, which limits the generalizability of the findings due to differences among races. Finally, it should be noted that although several covariates were controlled for during the study, other potential confounders, such as diet and hormone use, cannot be completely excluded.

VAI is a simple and easily accessible indicator of visceral fat function with good clinical applicability. Exploring the relationship between VAI and endometriosis could help screen high-risk populations in areas with limited medical resources. It can also provide the basis for developing individualized prevention and treatment programs. The results of this study suggested that VAI may be a valid indicator of endometriosis risk. Clinicians can advocate that controlling visceral fat accumulation (e.g., reducing trans-fat intake) may reduce the probability of developing the disease in high-risk populations. This study can only serve as a preliminary exploration of the risk of EMS in women with different degrees of VAI. Considering these limitations, further studies are necessary to validate clinical generalizability and explore the underlying mechanisms.

## 5 Conclusion

Overall, the study found that VAI was linked to the risk of endometriosis. However, more large-scale prospective studies are necessary for validation.

## Data Availability

Publicly available datasets were analyzed in this study. This data can be found here: https://wwwn.cdc.gov/nchs/nhanes/.
