# Supplementary material for: Association between visceral adiposity index and endometriosis: a population-based study
Source: Front Nutr. 2025 Jul 21;12:1602288. doi: 10.3389/fnut.2025.1602288 (PMC12318732; doi:10.3389/fnut.2025.1602288)
Supplement: Supplementary file 1 [file Table_1.docx]

**Supplementary material**

Table S1: Collinearity diagnostics steps.

|  | VIF |  |
| --- | --- | --- |
| VAI | 1.05 | |
| Age | 1.14 | |
| Race | 1.04 | |
| Education | 1.07 | |
| Marital status | 1.12 | |
| Poverty-to-income ratio | 1.10 | |
| Smoking | 1.09 | |
| Drinking | 1.09 | |
| Diabetes | 1.04 | |
| Hypertension | 1.07 | |
| Oral contraceptive | 1.03 | |
| Pregnancy | 1.15 | |
| Age at menarche | 1.01 | |
| Physical activity | 1.06 | |

VIF: variance inflation factor; VIF = 1/(1-R^2^)

Abbreviations are as follows. VAI: Visceral adiposity index

Note: The variables with VIF>10 will be regarded as collinear variables and cannot be included in the multiple regression model

Table S2. Association between BMI/WC and the risks of endometriosis.

|  | OR (95%CI), *p*-value | | |
| --- | --- | --- | --- |
|  | Crude model | Minimally adjusted model | Fully adjusted model |
|  | (Model 1) | (Model 2) | (Model 3) |
| BMI | 1.01 (0.99, 1.03),  *p* = 0.437 | 1.01 (0.99, 1.03),  *p =* 0.381 | 1.01(0.99, 1.03),  *p* = 0.347 |
| WC | 1.01 (1.00, 1.02),  *p* = 0.114 | 1.01 (1.00, 1.02)  *p* = 0.155 | 1.01 (1.00, 1.02)  *p* = 0.158 |

95% CI, 95% confidence interval; OR, odds ratio; BMI, Body mass index; WC, Waist circumference

Model 1 adjust for: None.

Model 2 adjust for: Age, Race, Education, Marital, PIR.

Model 3 adjust for: Age, Race, Education, Marital, PIR, Smoking, Drinking, Diabetes, Hypertension, Oral contraceptive, Pregnancy, Age at menarche, Physical activity.

Table S3. Sensitivity analyses.

|  | OR (95%CI), *p*-value | | |
| --- | --- | --- | --- |
|  | Crude model | Minimally adjusted model | Fully adjusted model |
|  | (Model 1) | (Model 2) | (Model 3) |
| VAI | 1.07 (1.03, 1.11)  *p* < 0.001 | 1.06 (1.02, 1.10)  *p* = 0.003 | 1.05 (1.01, 1.09)  *p* = 0.016 |
| VAI (quartile) | | | |
| Q1 | Reference | Reference | Reference |
| Q2 | 0.90 (0.51, 1.58)  *p* = 0.704 | 0.92 (0.53, 1.60)  *p* = 0.768 | 0.90 (0.51, 1.59)  *p* = 0.719 |
| Q3 | 1.22 (0.68, 2.18)  *p* = 0.493 | 1.23 (0.66, 2.29)  *p* = 0.505 | 1.09 (0.57, 2.08)  *p* = 0.790 |
| Q4 | 2.28 (1.37, 3.82)  *p* = 0.002 | 2.26 (1.35, 3.79)  *p* = 0.003 | 1.91 (1.12, 3.26)  *p* = 0.019 |
| *p* for trend | 0.002 | 0.002 | 0.015 |

95% CI, 95% confidence interval; OR, odds ratio.

Model 1 adjust for: None.

Model 2 adjust for: Age, Race, Education, Marital, PIR.

Model 3 adjust for: Age, Race, Education, Marital, PIR, Smoking, Drinking, Diabetes, Hypertension, Oral contraceptive, Pregnancy, Age at menarche, Physical activity.
